# Teachers Between Job Satisfaction and Burnout Syndrome: What Makes Difference in Czech Elementary Schools

**DOI:** 10.3389/fpsyg.2019.02287

**Published:** 2019-10-29

**Authors:** Irena Smetackova, Ida Viktorova, Veronika Pavlas Martanova, Anna Pachova, Veronika Francova, Stanislav Stech

**Affiliations:** Department of Psychology, Faculty of Education, Charles University, Prague, Czechia

**Keywords:** burnout syndrome, teachers, self-efficacy, social support, job satisfaction

## Abstract

As has been shown by several studies, teaching is a highly stressful occupation (Johnson et al., 2005), and most teachers experience work stress. Long-term stress decreases job satisfaction and can result in chronic exhaustion which can develop into burnout syndrome. Implications of burnout syndrome are strongly negative both for the personal and professional life of teachers. As burnout syndrome puts teachers’ well-being, quality of the teaching process and relationships with students at risk, it is important to seek ways to avoid teachersÉ’ burnout. Many studies have confirmed the protective role that coping strategies play in managing stressful situations, teacher’s self-efficacy or social support. In previous studies, a negative connection was found between burnout syndrome and job satisfaction. Job satisfaction is a crucial element in the definition of wellbeing. We find wellbeing rather not as a resource for burnout prevention but as an opposite state to burnout syndrome. The paper presents a quantitative survey on burnout syndrome and related variables among Czech elementary school teachers (*n* = 2,394). According to burnout score, two subgroups were selected – teachers with no burnout manifestations and teachers with developing/developed burnout syndrome. Through the statistical analysis, these two groups were compared in job satisfaction, self-efficacy, coping strategies, and social support. The study shows significant differences between the two groups of teachers in all examined variables. The strongest impact on burnout syndrome was found for negative coping.

## Introduction

As has been shown by several studies, teaching is a highly stressful occupation (Johnson et al., 2005). Most teachers experience work stress from stable distinctive conditions of teacher occupation and from specific demands related to technology and authority in the new era (Chan and Hui, 1995; Caprara et al., 2006). Stress comes when a person does not manage the task ahead. S/he has to put a lot of effort into mastering it. At the same time, s/he must also process the negative emotions they experiences due to failure (Lazarus, 1993; Hobfoll, 2002). Both for the first and second reasons, exposure to long-term stress leads to exhaustion. Long-term stress decreases job satisfaction and can result in chronic exhaustion which can develop into burnout syndrome (Leiter et al., 2014), especially in occupations with incessant human interactions and a high level of responsibility for others (Brouwers and Tomic, 2000; Johnson et al., 2005).

Burnout is included in the 11th Revision of the International Classification of Diseases (ICD-11) as an occupational phenomenon. WHO defines burnout syndrome as “resulting from chronic workplace stress that has not been successfully managed. It is characterized by three dimensions: (1) feelings of energy depletion or exhaustion; (2) increased the mental distance from one’s job, or feelings of negativism or cynicism related to one’s job; and (3) reduced professional efficacy. Burnout refers specifically to phenomena in the occupational context and should not be applied to describe experiences in other areas of life” (ICD-11).

Implications of burnout syndrome are strongly negative both for the personal and professional life of the teacher. Many studies confirm that teachers with high level of burnout syndrome are not able to establish positive relationships with students, to understand students’ needs, to stay in contact with trends in their field and to provide pedagogically effective lessons (Greenglass et al., 1996; Yong and Yue, 2007; Shen et al., 2015). Other studies show that teachers with a high level of burnout syndrome suffer from somatic problems such as back-pains or headache, from low self-esteem, a lack of meaningful-life orientation, interpersonal conflicts, and low social support (Hultell et al., 2013; Kidger et al., 2016). In many cases, burnout syndrome results in depression (Dilekmen and Erdem, 2013). It can result in a high rate of absences, dropouts and early retirements of burnout teachers, which increases costs (Brackett et al., 2010). As burnout syndrome puts teachers’ well-being, quality of the teaching process and relationships with students at risk, it is important to seek ways to avoid teachersÉ’ burnout. Loonstra et al. (2009) argue that teacher burnout is a barrier to a higher quality of the educational system and thus it is necessary to pay scientific and political attention to this.

The burnout syndrome can be considered as a diagnostic category for medical reasons, as an investigatory category for psychological or medical research or as a real-life category for an individual description of subjective state. Despite some theoretical problems referring to burnout syndrome as a diagnostic and investigatory category (Kaschka et al., 2011; Bianchi et al., 2015), we find this term very useful. In our experience and in some studies (Pyhältö et al., 2011), teachers often described their professional experience and feelings with using expressions such as lack of flame or no spark or out of battery, etc. Such expressions refer to burnout. Thus, it means that burnout syndrome represents an *in vivo* code. Conversely, teachers with a high level of energy and enthusiasm self-report being satisfied in their profession and even in their life (Skaalvik and Skaalvik, 2007, 2014). They use the following statements: I am still happy with students; it makes me feel self-realized; I find contentment in teaching, etc.

Based on our psychological experience and on literature (Van Horn et al., 2004; Pyhältö et al., 2011; Skaalvik and Skaalvik, 2014), we conclude that burnout syndrome stands in the opposition to the job satisfaction. However, we can replace job satisfaction with life satisfaction which is used in definitions of well-being (Diener and Suh, 1997). Diener and Suh (1997) state that “well-being consists of three interrelated components: life satisfaction, pleasant affect, and unpleasant affect. Affect refers to pleasant and unpleasant moods and emotions, whereas life satisfaction refers to a cognitive sense of satisfaction with life” (Diener and Suh, 1997, p. 200). Besides of life satisfaction, two other dimensions of well-being: the ability to fulfill goals and happiness (Pollard and Lee, 2003). Dodge et al. (2012) put in the center of well-being a state of equilibrium between resources and challenges in psychological, social and physical ways.

The theoretical background for our study covers not only the conceptualization of well-being by Dodge et al. (2012) but also the conservation of resources theory by Hobfoll (2002). This theory assumes that individuals seek to obtain, retain, and protect resources. Resources are socio-culturally influenced and consist not only of personality traits and social relationships but also of materials or life conditions (Hobfoll, 2002). Despite gaining resources that brings well-being, losing resources brings stress, including burnout (Grandey and Cropanzano, 1999). Based on the conservation of resources theory, the central motivation for individuals refers to the acquisition and facilitation of resources which is heightened especially in moments when resource loss occurs or are threatened. “Resources are seen as the essential elements of people’s stress resistance armamentarium, loss of resources tends to lead to resource loss cycles that have increased strength and speed.” (Hobfoll, 2002, p. 312). Individuals with a high level of resources are less likely to encounter stressful situations and thus their well-being is not negatively affected. The well-being of teachers is a widely studied field. Most studies find lower wellbeing scores of teachers when compared to working population samples (Johnson et al., 2005; Kidger et al., 2016).

There are three theoretical assumptions in our study which come from previous findings presented above: (1) the burnout syndrome is caused by chronic stress, (2) the stress results from threatened or lost resources that establish unbalance between resources and challenges, (3) the balance between resources and challenges is a fundamental feature of well-being. Based both on research findings and on our psychological experience, we postulate burnout syndrome and well-being as the opposites. If teachers report high burnout, they do not report high well-being and vice versa. This postulation brings us to questioning differences between teachers with a high level of burnout syndrome and with a high level of well-being. The goal of the presented study is to compare those two groups of teachers to find out in which characteristics they differ. Some of these differences can be a field for the prevention of burnout syndrome among teachers. The prevention of burnout syndrome makes a frame of our study because we argue similarly to Loonstra et al. (2009) that teacher burnout is a barrier for the higher quality of the educational system.

Burnout prevention has been studied for more than three decades. During this period, the scientific attention has been moved from demographic characteristics, like gender or age, to more subtle and psychological relevant characteristics. The latest research findings confirm the protective role of positive, problem-centered coping strategies (Austin et al., 2005), strong professional self-efficacy (Skaalvik and Skaalvik, 2007) and collegial social support (Martin and Dowson, 2009; Buchwald, 2013).

Coping strategies are defined as the patterns of an individual’s responses to stress, which remains relatively stable – either over time or in different situations (Frydenberg, 2008) and as the degree to which an individual is able to manage stress experienced in certain situations (Brouwers and Tomic, 2000). Most authors agree that some coping strategies help to avoid stress-producing situations due to changing the source of stress, while others do not because they are focusing just on the actual regulation of negative emotions (Tobin et al., 1989; Lazarus, 1993; Kyriacou, 2001; Marroquin et al., 2010). In our study, the typology by Janke and Erdmann (2003) was used. It distinguishes six coping strategies as positive strategies because they reduce stress (play down, guilt denial, substitutional satisfaction, situation control, reaction control, and positive self-instruction); four are considered negative strategies because they do not remove the source of stress and thus the level of stress increases over the long-term (flight tendency, rumination, resignation, and self-accusation). Two strategies are characterized as neutral because their effects are ambivalent and depend on the situational context (need for social support and active avoidance). Kepalaite (2013) found that teachers who used positive reappraisal (e.g., situation control) were inclined to use other positive strategies more often as well (e.g., positive self-instruction); and vice versa, teachers who used negative strategies (e.g., escape) also used other negative strategies more often (e.g., distancing). Studies by Chwalisz et al. (1992), Austin et al. (2005), Marroquin et al. (2010), and Shin et al. (2014) show that negative strategies are associated with higher burnout levels.

Teacher self-efficacy is defined as the set of beliefs that teachers hold about their professional competencies and their efficiency to plan, organize, and carry out different educational activities and to solve challenging situations (Guskey and Passaro, 1994; Brouwers and Tomic, 2000; Bandura, 2006; Skaalvik and Skaalvik, 2014). Guskey and Passaro (1994) emphasize that teachers with high self-efficacy believe in their personal influence, power, and impact on the educational process, including students’ learning (internal dimension) and factors outside the classroom (external dimension). Several studies have confirmed a moderate negative correlation between teacher self-efficacy and burnout (Brouwers and Tomic, 2000; Skaalvik and Skaalvik, 2007).

Social support at the workplace is defined as the set of activities by colleagues, managers, and leaders which are interpreted as helpful or intended to be helpful (Deelstra et al., 2003; Harris et al., 2007). Social support covers a variety of interpersonal behaviors among colleagues (such as providing emotional support, assisting, mentoring, etc.) that enhance the psychological or behavioral functioning of individual workers. Most studies find social support at the workplace to be positively predictive of job satisfaction and well-being (Harris et al., 2007) and negatively predictive of burnout syndrome (Myung-Yong and Harrison, 1998).

Earlier studies have confirmed the positive effects of each characteristic described above on the prevention of teacher burnout syndrome. Teachers reporting high self-efficacy scored lower in burnout scale (Skaalvik and Skaalvik, 2007, 2014), teachers reporting positive, problem-centered coping strategies score lower in the burnout scale (Austin et al., 2005) and teachers reporting strong workplace social support score lower on the burnout scale (Harris et al., 2007). However, there is still a lack of empirical evidence about mutual relationships between all three variables among teachers with burnout syndrome and with well-being.

With regard to previous knowledge in the field, our study attempts to find out what relationships between burnout syndrome on the one hand and self-efficacy, coping strategies, social support, and job satisfaction on the other hand exist. The overall aim of the presented study focuses on a comparison between teachers with a high and low score in burnout syndrome in the following four dimensions: self-efficacy, coping strategies, social support, and job satisfaction. We assume that teachers without burnout syndrome have stronger self-efficacy (hypothesis 1), use more often positive coping strategies (hypothesis 2) and get better workplace social support from colleagues and principals (hypothesis 3). Because of opposition between burnout syndrome and wellbeing, we assume that teachers with developing or developed burnout syndrome feel more often unsatisfied in their profession (hypothesis 4).

## Materials and Methods

The study has quantitative correlation design using self-report questionnaires within cross-sectional approach. The study was conducted among elementary school teachers in the Czechia. The Czech educational system is specific in three ways (Education at a Glance, 2016; Ministry of Education, 2017): in its organization (primary and lower-secondary educational levels are combined in elementary schools; however, specific university training is required for each level), in its financing (schools have been underfinanced for many years and teachers’ salaries are roughly equal to the national average but significantly below the national average for university graduates), and in a gradual deterioration of student achievement (as indicated by TIMMS and PISA tests).

### Participants

Teachers were asked to participate in the study via an email distributed to all elementary school principals in the Czechia and via educational journals. The sample consisted of teachers who were willing to participate in the research, which may introduce a certain bias. However, a comparison of all teachers working in elementary schools in the Czechia in 2016 (according to 2016 Ministry of Education statistics) showed that the sample used in this study was representative in terms of gender and educational levels. An online version of the questionnaire was made available to respondents for completion for 13 weeks during the winter of 2016 and spring of 2017. All respondents were informed about the aims of the study and the ways how the results will become public. They were also ensured that their answers will be anonymized. The ethical guidelines were followed both in collecting and analyzing of data.

The study included 2,394 teachers who work in Czech elementary schools. The set of respondents consisted of 358 male teachers (15%) and 2,036 female teachers (85%). The average number of years of teaching experience was 21.42 (*SD* = 10.67). Czech elementary schools operate on two educational levels: the primary level is for children from 6 to 11 years of age and the lower-secondary level for students from 11 to 15 years. Each educational level requires specific teacher training. The sample included 976 primary education teachers (41%), 859 lower-secondary education teachers (36%), and 559 teachers specializing in both levels (23%).

Using burnout scale, the sample was divided in three sub-groups: teachers with no burnout (*n* = 383), teachers at risk of burnout (*n* = 1,545) and teachers with developing or developed burnout (*n* = 464). In the analyses, sub-groups with no manifestation and with strong manifestation of burnout were compared. This groups were similar in terms of age, length of teaching practice, teacher training and school size (no significant difference was found in these variables).

### Instruments

Burnout was measured using the Shirom–Melamed Burnout Scale (Shirom and Melamed, 2006) which had been validated for the Czech population. The Shirom–Melamed Burnout Scale conceptualizes burnout along three dimensions: physical fatigue, emotional exhaustion and cognitive weariness. The SMBM is comprised of 14 items divided into three sub-scales. The examples of particular items are: *I have no energy for going to work in the morning; I feel like my “batteries” are “dead”; I feel I’m not focused in my thinking.* Each sub-scale consists of several items evaluated on a 7-point Likert scale ranging from “*never or almost never*” to “*always or almost always.*” High summary scores are indicative of burnout. The reliability measured by Cronbach’s alpha was 0.939. The factor structure of the 13-item SMBS was explored by means of exploratory factor analysis with Varimax rotation and eigenvalues greater than 1. The analysis extracted six factors that were consistent with the theoretical SMBS model. These factors explained 81% of the variance in the equation. The expected factor loadings were greater than 0.7 for all items. Thus, the SMBS proved to be an excellent tool for measuring the sample’s burnout.

Coping strategies were measured by the Stress Coping Style Questionnaire (SVF 78) by Janke and Erdmann (2003) which had been standardized for the Czech population. The measurements were based on the following assumptions. The questionnaire used a common introductory sentence: *When someone upsets me, something disturbs me, or I am thrown off balance in some way*, … Each of the 78 items completes this sentence, for example: *I tell myself*, “*It’s not that bad.*” The respondent self-evaluates each assertion based on the probability of his/her reaction using a 5-point scale ranging from “*not at all*” to “*very likely.*” The 78 items are divided into 12 sub-scales representing the following ways of reacting to stressful situations: play down, guilt denial, substitutional satisfaction, situation control, reaction control, positive self-instruction, need for social support, active avoidance, flight tendency, rumination, resignation, and self-accusation. To interpret questionnaire scores, Janke and Erdmann (2003) distinguish between positive coping strategies (six sub-scales), negative coping strategies (four sub-scales) and two neutral coping strategies. In our study, the reliability of the SVF78 measured by Cronbach’s alpha was 0.89, which is satisfactory.

Teacher self-efficacy was measured using the Czech Teacher Self-Efficacy Scale (CTSES; Smetáčková et al., 2017) which was developed specifically for the Czech educational system per Bandura’s guidelines (Bandura, 2006). The CTSES includes 45 items measured on a 5-point scale ranging from “*never*” to “*always.*” Each item starts with a formulation: “*I am convinced that I can*…” and the particular professional skill follows, for example: “*adapt school tasks and instructions to be clear for all children*”; “*cooperate with other teachers on making educational process more effective.*” The reliability of the scale measured by Cronbach’s alpha was 0.948. The CTSES is comprised of six sub-scales: pedagogical approach, student diversity, collaboration with parents, discipline, influence on school management, cooperation among teachers, and professional development.

Social support was measured by a newly created Scale of Perceived Quality of Workplace Relationships that contained eight statements (e.g., *At school I have people with whom I can deal with my work difficulties*), with whom they expressed their agreement on a four-point scale from “definitely.” Scale reliability was 0.8 (measured by Cronbach’s alpha).

Job satisfaction was measured by a battery of items focusing on 16 aspects of teacher occupation such as relationships with colleagues, relationships with management, students, curricula, Ministry of Education etc. Respondents were asked: *In which level are you satisfied with each of following areas of your profession?* The level of satisfaction was expressed on a four-point scale from “very satisfied” to “very dissatisfied.” Scale reliability was 0.85 (measured by Cronbach’s alpha).

### Data Analysis

Data were analyzed using IBM SPSS analytics software. In the first step, the collected data were mapped to the CTSES, the Shirom–Melamed Burnout Scale, the SVF78, the Scale of Perceived Quality of Workplace Relationships and the Job Satisfaction Scale. In all five cases, the structuring of the data allowed for the use of parametric tests. We also measured reliability using Cronbach’s alpha to verify that the scales had the same validity for this sample as for other studies. In the second step, mean values on all scales were determined including total mean values, sub-scale mean values and mean values in each sub-group. Differences between the mean values were determined through a t-test. In the third step, correlations for all three scales and their sub-scales were analyzed. In the last step, we performed a hierarchical regression analysis to establish the statistical significance of relationships among variables. In all particular steps, we compare two groups of respondents – teachers with high scores in the burnout scale (corresponding to present, a serious and very serious manifestation of burnout syndrome based on the SMBM norms) and teachers without burnout symptoms (corresponding to the total lack of manifestation of burnout).

## Results

In the first step, descriptive statistics for all variables were computed to explore and describe characteristics of Czech teachers. The results are shown in Table 1. On average, teachers reported mild burnout. No burnout was reported by 16% of teachers, very mild burnout by 32% of teachers, mild burnout by 33% of teachers, present burnout by 15% of teachers, serious burnout by 4% of teachers and very serious burnout by 1% of teachers. There was no gender gap in the total scores. The strongest burnout was reported on the physical scale, with milder burnout on the cognitive scale and the lowest on the emotional scale. While male teachers reported stronger emotional burnout, female teachers reported stronger physical burnout.

**TABLE 1:** Means, standard deviations, minimum, and maximum (*N* = 2,394).

	**Mean**	**SD**	**Scale Min–Max**
Burnout syndrome	40.89	13.679	13; 91
Self-efficacy	166.95	18.913	45; 225
Positive coping strategies	13.56	2.609	0; 25
Negative coping strategies	11.38	3.789	0; 25
Social support	13.85	3.884	8; 32
Job satisfaction	2.41	0.346	1; 4

The results showed that teachers used positive coping strategies more often than negative coping strategies. The most common coping strategy was substantial gratification, which belongs to positive strategies. An often used strategy was also searching for social support which belongs to neutral strategies. As an independent variable, the workplace social support was found to be also rather higher. Most teachers reported also high satisfaction with relationships with colleagues and principals. The job satisfaction, in general, was moderately high, especially in aspects related to school characteristics instead of educational system characteristics.

With regard to self-efficacy, the study showed that Czech teachers have moderately weak self-efficacy, contrary to our expectations. Most teachers perceived themselves as professionally competent sometimes or rarely. The highest level of self-efficacy was reported in the dimensions of Collaboration with parents and Professional development. On the contrary, the lowest self-efficacy was reported in the Pedagogical approach, which is considered to be a crucial aspect of teaching beliefs. Thus, these findings have important consequences for at-risk aspects of teacher identity.

In the second step, the mutual relationships among examined characteristics were measured. Table 2 shows correlations between the study variables. All correlations with burnout syndrome were strongly significant. However, the correlation with social support was too weak and thus probably ecologically invalid. Correlations with three other variables were sufficiently strong both in terms of statistic findings and in reality. The results confirm that burnout syndrome is lower when teachers have strong self-efficacy, feel satisfied, use positive coping and avoid negative coping, and vice versa.

**TABLE 2:** Bivariate correlations (*N* = 2,394).

		**Self-**	**Positive**	**Negative**	**Social**	**Job**
	**Burnout**	**efficacy**	**coping**	**coping**	**support**	**satisfaction**
Burnout		–0.293^∗∗^	–0.259^∗∗^	0.553^∗∗^	0.068^∗∗^	0.354^∗∗^
Self-efficacy			0.354^∗∗^	–0.214^∗∗^	–0.058^∗∗^	–0.207^∗∗^
Positive coping				–0.291^∗∗^	–0.033	–0.104^∗∗^
Negative coping					0.039	0.186^∗∗^
Social support						0.089^∗∗^
Job satisfaction						

*Correlations were significant, ^∗∗^*p* < 0.001.*

The correlation between teacher burnout syndrome and teacher self-efficacy was −0.293. Teachers with high self-efficacy scored low in burnout symptoms and vice versa. All three dimensions of burnout showed a significant negative correlation with teacher self-efficacy. However, the strongest value was reported for the emotional dimension (−0.375). Teachers with high self-efficacy scored low in emotional burnout, which could have the most negative impact on relationships between teachers and students and thus on student well-being in schools.

Burnout syndrome was strongly correlated to both types of coping strategies. The correlation between positive coping and burnout symptoms was 0.259 and the correlation between negative coping and burnout symptoms was even as high as 0.553. The more teachers used negative coping strategies, the higher they scored for burnout symptoms. The more teachers used positive coping strategies, the lower they scored for burnout symptoms. Positive and negative coping strategies are not mutually exclusive; a person can use both positive and negative coping strategies during the same period of his/her life. However, a negative correlation was found between these two types of coping strategies (−0.291). This implies that most teachers tend to use only one coping style.

A significant correlation between both coping styles and teacher self-efficacy was found as well. Positive coping correlates positively (0.354) and negative coping correlates negatively (−0.214). This means that teachers with high self-efficacy score high in positive coping but rather low in negative coping. All sub-scales of teacher self-efficacy show similar patterns.

Moreover, a significant correlation between job satisfaction and all other variables was found. Teachers with higher job satisfaction reported not only lower burnout syndrome, but also stronger self-efficacy, more frequent use of positive coping and avoiding of negative coping and receiving better social support (although, this relation was relatively weak).

In the third step, respondents were divided into three subgroups according to global score in the Shirom–Melamed Burnout Measurement. The norms indicated teachers with no burnout manifestation and five more levels of increasing burnout manifestations. The three highest levels – presented, serious and very serious burnout manifestations – were put together and established the sub-group called “developing and develop burnout.” Two subgroups with no burnout and with existing burnout were compared in the following statistical analyses. The group with no burnout reported the global score *M* = 21.45 (*SD* = 3.588), while the group with developing/developed burnout syndrome reported the global score *M* = 60.89 (*SD* = 7.502). The gap between global scores is significant, *p* < 0.001. In the following steps, the variables between identified subgroups were compared. The means and their statistical significance are shown in Table 3.

**TABLE 3:** Means, standard deviations, and significance of difference (*N* = 847).

	**No burnout**	**Burnout**	***p*-value**
Self-efficacy	177.31 (18.922)	160.75 (20.690)	<0.001
Positive coping strategies	14.59 (3.032)	12.56 (2.566)	<0.001
Negative coping strategies	8.41 (3.423)	14.53 (3.233)	<0.001
Social support	13.19 (3.533)	14.11 (4.098)	<0.001
Job satisfaction	2.23 (0.358)	2.62 (0.328)	<0.001

There is a significant difference between burnout and non-burnout teachers in all variables. Teachers with high score in burnout scale have lower job satisfaction, receive lower social support at workplace, report lower self-efficacy, use less often positive coping strategies and use more often negative coping strategies.

In the fourth step, in order to determine which variable shows a higher degree of burnout predictability, multiple linear regression was conducted using SPSS software. In a model with the burnout level as a dependent variable, total self-efficacy scores, positive coping, negative coping, social support and job satisfaction were tested in a regression equation as independent variables. All details are presented in Table 4.

**TABLE 4:** Hierarchical regression analysis for predicting burnout syndrome.

**Predicting variable**	**β**	**Δ*R*^2^**	***F***	***p***
**Model 1**				
		0.388	246.41	<0.001
Self-efficacy	–0.126	–0.092	–6.771	<0.001
Positive coping	–0.051	–0.264	–2.690	<0.007
Negative coping	0.467	1.697	25.581	<0.001
Social support	0.026	0.092	1.525	<0.127
Job satisfaction	0.230	9.071	12.994	<0.001

The results of the hierarchical regression analysis showed that four variables in the model were significantly related to burnout symptoms. The only variable without significant link was Social support. Teachers’ burnout level decreased by 2.30 points for each standard score of total job satisfaction, by 0.13 point of each standard score of total self-efficacy, by 0.05 point for each standard score of total positive coping, while the burnout level increased by 0.467 points for each standard score of negative coping.

The same model was tested separately for subgroups of teachers with no burnout and with developing/developed burnout. *R*^2^ value was 0.037 for no-burnout-group and 0.109 for burnout group. A significant degree of burnout predictability was found only for self-efficacy in the no-burnout-group (*p* < 0.046), while for negative coping (*p* < 0.001) and job satisfaction (*p* < 0.024) in the burnout-group.

## Discussion

The aim of the study was to test whether there is a relationship between teacher burnout syndrome, self-efficacy, coping strategies, workplace social support, and job satisfaction. In our study, we apply a perspective of the conservation of resources theory by Hobfoll (2002) and the perspective of wellbeing as a balance between resources and challenges (Dodge et al., 2012). Based on these theories, we search for professional resources which can teachers use to preserve their pedagogical energy and competencies. In the listed variables, we have searched for differences or accordance with teachers with a high level of burnout symptoms and without any burnout symptoms.

Earlier studies have found strong evidence for a connection between burnout syndrome and self-efficacy (Brouwers and Tomic, 2000; Skaalvik and Skaalvik, 2007), for a connection between burnout syndrome and coping style (Chan and Hui, 1995) and for a connection between burnout syndrome and social support (Myung-Yong and Harrison, 1998). Some studies also found evidence for strong protective function of job satisfaction to burnout syndrome (Harris et al., 2007). However, connections between all these variables have not been analyzed sufficiently to date. The lack of empirical findings exists from educational systems out of United States and Western Europe. Current knowledge about prevention of burnout syndrome and promoting teacher wellbeing need to be completed and compared with research from other countries, like the Czechia as post-communist country. Every educational system, with its specific values and organizational structure, introduces different challenges and accepted or rejected ways of responding to them (Chan and Hui, 1995). The Czechia as a new research location in this field can be therefore taken as a validation of previous findings on the burnout prevention. The presented study results in the conclusion that examined variables as concepts universally exist, however, the ways how they are manifested and how they can be measured are partially culture-dependent (Bandura, 2006).

Our study worked with four hypotheses derived from previous research. We expected that teachers with developing or developed burnout syndrome have stronger self-efficacy (hypothesis 1), use more often positive coping strategies (hypothesis 2) and get better workplace social support from colleagues and principals (hypothesis 3). With regards to the opposition between burnout syndrome and wellbeing, we assume that teachers with developing or developed burnout syndrome feel more often unsatisfied in their profession (hypothesis 4). All four hypotheses were confirmed.

Our study has confirmed that there is a close relationship between burnout and job satisfaction and also self-efficacy, coping strategies and social support among teachers. Teachers with lower burnout scores reported stronger self-efficacy, more frequent using of positive coping strategies, avoiding negative coping strategies and better workplace social support. This result is relevant for the conservation of resources theory (Hobfoll, 2002) in rationalizing how specific psychological resources may shield teachers against the hazards of work burnout.

Most findings from our study were in correspondence with previous studies on self-efficacy. Austin et al. (2005) found that teachers who reported lower stress levels used positive coping more often, while teachers who reported higher stress levels used negative coping more often. Skaalvik and Skaalvik (2007, 2014) showed that teachers with high self-efficacy scored lower on burnout symptoms and vice versa. Brouwers and Tomic (2000) reported that teacher self-efficacy was significantly related to their burnout levels, however, only with respect to the depersonalization and personal-accomplishment dimensions of burnout, and not with respect to the emotional-exhaustion dimension. In our study, all three dimensions of burnout syndrome – physical, emotional, and cognitive – were significantly related both to self-efficacy and to coping strategies.

Also, our study is in agreement with previous findings on coping strategies. Studies by Chwalisz et al. (1992), Austin et al. (2005), Marroquin et al. (2010), and Shin et al. (2014) showed that using positive, problem-centered coping is linked to lower score in burnout, while using negative, emotion-centered coping is linked to higher score in burnout. However, based on these studies, we assumed that protective function of positive coping is stronger than inhibition caused by negative coping. Our study showed that the connection between burnout and negative coping was stronger. It means that the avoidance of negative coping helps in prevention against burnout syndrome more effective than using positive coping.

Many previous studies showed that social support at workplace had positive effect for job satisfaction and for prevention of burnout syndrome (Harris et al., 2007; Leiter et al., 2014). Our study confirms this result. However, the connection between social support and burnout syndrome was not as strong as we expected. The social support does not have a predictability degree for burnout score in either the no-burnout-group nor burnout-group. In a separate analysis, the significant predictability showed only self-efficacy for teachers without any burnout symptoms and job satisfaction and negative coping for teachers with developing/developed burnout. We find this finding challenging, and encourage further studies in this field because it has a high relevance for supporting teachers’ wellbeing.

The presented study has several limitations. One limitation of this study is that we measured only four variables, however, there are other dimensions of the school and individual context (personal traits or collective efficacy e.g.) which should be analyzed in future research. As another limitation to be considered is the fact that the study was conducted just in the Czechia. However, it is not only the national study with a regional contribution. Czech teachers had not previously participated in any international research about this issue. The Czech educational system is different from systems in the United States, Western Europe, and Scandinavia, where the majority of research has been conducted. This study in the Czech as a new research location in this field can be therefore taken as a validation of previous findings on the correlation between burnout and self-efficacy and burnout and coping strategies. The presented study results in the conclusion that these concepts universally exist, however, the ways how these concepts are manifested and how they can be measured are culture-dependent (Bandura, 2006).

Cultural dependency was the reason for the development of the CTSES. However, using a new instrument to measure the self-efficacy which has been yet not compared to other measurements on self-efficacy can be considered as the third limitation of the study. Therefore, it is not known whether the level of self-efficacy among Czech teachers is higher or lower than in other countries. The advantage is that the instrument measuring the self-efficacy specifically addressed teacher occupation. Conversely, the measurement of coping strategies is general and does not respect the school environment. The using of specific measurement of teacher coping strategies would be helpful in future research. We used the SVF78 to measure coping strategies of teachers, although this instrument is too general. For example, distancing represents an effort to break away from a situation and not take it seriously, which may be a functional strategy in situations where an individual has no real control over the problem. However, in the case of teaching, this strategy results in an unprofessional approach where students, their needs and the context in which the education process takes place are ignored. As our study showed, it is important to imply the perspectives specific to certain professions, in our case teaching. We consider future development of a specific measurement for teachers’ coping strategies important, especial for Central European educational systems which differ significantly from school systems in the United States and other countries.

## Author’s Note

The research was conducted by a team of psychologists from the Charles University, Prague, Czechia, namely: IS, IV, VP, VF, AP, SS, Radek Ptacek, Petra Topkova, and Jiri Raboch.

## Data Availability Statement

The datasets generated for this study are available on request to the corresponding author.

## Ethics Statement

Ethics approval was not required as per the local legislation and national guidelines.

## Author Contributions

All authors listed have made a substantial, direct and intellectual contribution to the work, and approved it for publication.

## Conflict of Interest

The authors declare that the research was conducted in the absence of any commercial or financial relationships that could be construed as a potential conflict of interest.
